# Evaluating buprenorphine to treat cancer pain: a systematic review protocol

**DOI:** 10.1186/s13643-025-02957-2

**Published:** 2025-11-10

**Authors:** Rafina Khateeb, Victoria Powell, Jack Rosenberg, Lauren Rose, Kayla Sheehan, Christian Mackey, Philip Papaioannou, Maria Silveira

**Affiliations:** 1 Division of Geriatric and Palliative Medicine, University of Michigan, F7896 UH South, SPC 5233, 1500 E. Medical Center Dr., Ann Arbor, MI 48109 USA; 2 Division of Hospital Medicine, University of Michigan, F4323 UH South, SPC 5352, 1500 E Medical Center Dr., Ann Arbor, MI 48109 USA; 3 Geriatrics Research Education and Clinical Center, LTC Charles S. Kettles Veteran Affairs Medical Center, 2215 Fuller Road, Ann Arbor, MI 48105 USA; 4 Department of Anesthesiology, University of Michigan, 1H247 UH, SPC 5048, 1500 E. Medical Center Dr., Ann Arbor, MI 48109 USA; 5 Department of Anesthesiology, LTC Charles S. Kettles Veteran Affairs Medical Center, 2215 Fuller Road, Ann Arbor, MI 48105 USA; 6 Hospice and Palliative Medicine Fellowship Program, The Ohio State University, 1581 Dodd Drive, Columbus, OH 43210 USA; 7 Department of Family Medicine, University of Michigan, 300 North Ingalls Street, NI4C06, Ann Arbor, MI 48109 USA; 8 Department of Family and Community Medicine’s Residency Program, University of New Mexico, 915 Camino de Salud NE, Albuquerque, NM 87106 USA; 9 Internal Medicine Residency Program, University of Michigan, 3116 Taubman Center, SPC 5368, 1500 E. Medical Center Drive, Ann Arbor, MI 48109 USA

**Keywords:** Cancer pain, Buprenorphine, Systematic review, Palliative care

## Abstract

**Background:**

Many patients with cancer experience pain related to their disease. Opioids are commonly used for moderate-to-severe cancer-related pain but carry significant side effects that may worsen quality of life. Buprenorphine, a partial mu opioid receptor (MOR) agonist, is increasingly used for the management of cancer-related pain. Although well studied as a treatment for opioid use disorder, its role in managing cancer pain is less understood.

**Methods:**

We aim to systematically gather, assess, and summarize prior studies on the impact of buprenorphine upon cancer-related pain. Cochrane CENTRAL, OVID Medline, EMBASE, EBSCO, and Web of Science will be searched in September 2025 for any studies available to date. Studies involving buprenorphine as an intervention upon cancer-related pain will be included where pain outcomes are measured at two or more timepoints using a validated scale. Exclusions apply to case studies/series with fewer than five subjects, qualitative studies, conference abstracts, review articles, research protocols, studies solely focused on post-operative pain, and studies not available in English translation. After final selection, study type classification, and data extraction, a quality assessment will be conducted using the Cochrane ROB 2 tool for randomized controlled trials (RCTs) and the Newcastle-Ottawa Scale for cohort and case-control studies. The summarized data will be evaluated using Grading of Recommendations Assessment, Development and Evaluation (GRADE) criteria.

**Discussion:**

The primary objective is to fill a knowledge gap by summarizing literature on buprenorphine for cancer pain management, including a broad range of study designs, drug formulations, and dosing. This systematic review aims to provide a comprehensive understanding of buprenorphine’s efficacy and safety in cancer pain management, which could inform clinical practice and future research directions.

**Systematic review registration:**

This protocol was registered in PROSPERO (CRD42022383591).

**Supplementary Information:**

The online version contains supplementary material available at 10.1186/s13643-025-02957-2.

## Background

Global cancer burden is growing according to the World Health Organization [1]. In 2022, there were an estimated 20 million new cancer cases [1]. More than half of adults receiving cancer treatment report experiencing pain related to cancer or its treatment [2]. Furthermore, two-thirds of those with advanced malignant disease experience pain [3, 4]. The etiology of cancer-related pain can include the disease itself (e.g., tumor growth and tissue invasion) or cancer treatment sequelae (e.g., chronic post-operative pain, chemotherapy-induced neuropathic pain) [5, 6]. Inadequately palliated cancer-related pain can severely impair quality of life, leading to emotional distress, reduced physical functioning, and social isolation [7–9]. Furthermore, inadequate pain management can increase healthcare utilization and costs [10]. Guidelines continue to recommend opioid analgesics for moderate-to-severe pain related to cancer unless contraindicated [11]. While full mu opioid receptor (MOR) agonists (e.g., morphine, fentanyl, and oxycodone) are commonly used to manage cancer-related pain, they have well-documented side effects and risks. In the short term, these include gastrointestinal symptoms (e.g., constipation, nausea), sedation or somnolence, accidental overdose, and respiratory suppression or arrest [12]. When used chronically, additional risks include hyperalgesia, tolerance, dependence, or misuse, highlighting the need for alternative therapies [13].

Buprenorphine, a partial MOR agonist commonly used to treat opioid use disorder (OUD), has recently emerged as a promising alternative to full MOR agonists in managing chronic pain requiring long-term opioids [14]. In certain subgroups of patients with chronic pain, buprenorphine may be particularly beneficial for mitigating opioid-related harms while maintaining analgesia. These include patients who demonstrate some aberrant opioid-related behaviors but do not fulfill criteria for diagnosis of OUD and those who experience intolerable side effects from other opioids [15, 16]. Accordingly, buprenorphine is experiencing a resurgence of clinical use for management of cancer-related pain in cancer patients with similar risks [17, 18]. The use of buprenorphine in treating OUD and chronic pain is well-supported [19, 20]. However, its role in cancer pain management is less understood, with limited high-quality evidence to guide clinical practice in this specific context. Some have argued that buprenorphine should be a first line for cancer pain [21]; however, a prior systematic review that examined the effectiveness of buprenorphine for cancer pain included data from randomized controlled trials (RCT) only, and its results did not find sufficient evidence to prioritize buprenorphine over full MOR agonists [22]. Data was equivocal regarding how buprenorphine compared to full MOR agonists with respect to cancer pain control, safety, and side effects/tolerability [22]. Not only were study sample sizes small, but there was substantial heterogeneity in study design, comparator groups, and drug formulation and dosing, limiting authors’ ability to draw conclusions [22]. Since the publication of this systematic review, there has been a rapid uptake in the clinical use of buprenorphine, including use as a first-line agent in managing cancer-related pain despite the lack of evidence in this unique population. Given the limitations of RCTs identified by the Cochrane authors and shifts in clinical practice patterns, a comprehensive systematic review is urgently needed.

We intend to conduct a systematic review to not only update previous review findings to include RCTs published since 2015, but also expand the scope of evidence to include observational and other study designs. Updating and expanding this review will help identify future research priorities and may ultimately inform clinical practice of cancer pain management.

## Methods/design

### Aim

To systematically gather, assess, and summarize literature examining the effectiveness of buprenorphine for pain related to cancer or its treatment, as well as buprenorphine’s safety, tolerability, and side effect profile in this population.

### Design

The systematic review will be conducted in accordance with the Cochrane handbook for systematic reviews of interventions [23]. This protocol is also reported according to the PRISMA-P checklist (see Supp File).

### Study eligibility

Study inclusion criteria will be informed by the PICOTS framework (see Appendix I).

Participants: The population of interest includes patients of any age with an active cancer diagnosis. Studies enrolling participants both with and without cancer diagnoses will be included insofar as they report pain data for the cancer subgroup.

Intervention: The intervention is buprenorphine in any formulation or dose.

Comparator: Comparators will include active controls (e.g., other opioids or other analgesics), placebo, or no comparators.

Outcomes: The primary outcomes to be assessed include pain intensity/severity and pain interference. Timing of measurements may vary; studies need to assess pain outcomes at least once before the intervention (e.g., baseline) and once during or after, using a validated pain assessment measure, to be included. Secondary outcomes include use of breakthrough medication, opioid dose reduction, side effects (including those associated with opioid withdrawal), adverse events, mental health (anxiety, depression, suicidality), quality of life, functional status, life expectancy, illicit drug use, and healthcare utilization where available.

Types of studies: RCTs, controlled observational (including case-control and cohort designs), and uncontrolled observational studies will be included. We will exclude case studies/series with fewer than 5 subjects, qualitative studies, conference abstracts (unless they are followed by a research publication), review articles, research protocols, and studies that examine only post-operative pain. We will seek English translations for all references in a foreign language, employing the assistance of professional librarians at the University of Michigan; in the instance when an English translation of the entire text cannot be found, the reference will be excluded.

### Search and selection

Cochrane CENTRAL, OVID Medline, EMBASE, EBSCO, and Web of Science will be searched in September 2025 for any studies available to date. Studies examining buprenorphine as an intervention for any type of pain (including nociceptive, neuropathic, nociplastic, and complex) in patients of any age with any kind of cancer (including hematologic malignancies) will be considered. Search terms will include terms used in the 2015 Cochrane review with modifications (see Appendix II). Citations will be imported into EndNote 21 (Thomson Reuters, NY, NY) for deduplication, then exported into DistillerSR [24] for screening, data extraction, and analysis of quality and risk of bias.

Study eligibility assessment will proceed through two levels using DistillerSR [24]. In level 1, two reviewers will independently examine study titles and abstracts against the inclusion criteria. In level 2, two reviewers will independently examine full text articles of selected studies from level 1 to confirm eligibility. If reviewers disagree at either screening level, conflicts will be resolved through discussion during study team meetings. The results of this screening and inclusion/exclusion process will be reported in full in the final systematic review manuscript utilizing a PRISMA flow diagram [25].

### Data extraction, quality assessment and synthesis

References meeting eligibility criteria from levels 1 and 2 above will enter level 3 data extraction. Using DistillerSR [24], two reviewers will extract information independently of each other regarding study design, setting, sample size, population, age and gender distribution, cancer characteristics (e.g., stage, type), baseline pain characteristics (severity, type), subject baseline opioid exposure, details of buprenorphine (e.g., formulation, dose, and frequency), frequency of adverse events, and study length. We will also extract data on the following outcomes when they are reported: anxiety, depression, physical function, health care use, illicit drug use, opioid dose reduction, pain interference, side effects (including those associated with opioid withdrawal), suicidality, use of breakthrough medication, and quality of life.

Studies will be classified as RCTs, controlled observational (including case-control and cohort designs), and uncontrolled observational studies. We plan to systematically review and summarize our findings into tables and descriptive text without quantitative data synthesis.

Study quality and risk of bias will be assessed in DistillerSR [24] by experienced reviewers in level 4 using the Cochrane ROB 2 tool for RCTs [26] and the Newcastle-Ottawa Scale for cohort and case-control studies [27]. Uncontrolled observational studies, such as pre-post designs, will be assumed to have an inherently high risk of bias. The strength of data will be assessed using the Grading of Recommendations Assessment, Development and Evaluation (GRADE) criteria [28] for each outcome of interest by the research team in an iterative fashion.

This protocol was registered in PROSPERO (CRD42022383591). Due to the nature of the study being a systematic review, it was exempt from IRB review and there was no active patient or public involvement in setting the research agenda. Any changes to this protocol will be included explicitly in the future systematic review manuscript.

## Discussion

Buprenorphine is an increasingly popular alternative to traditional full MOR agonists (e.g., morphine, oxycodone, fentanyl) for managing cancer-related pain, primarily due to clinician perceptions of a superior side effect profile and safety profile [21]. In patients with chronic, non-malignant pain who use long-term opioids, buprenorphine may maintain analgesia while mitigating the risks associated with long-term use including tolerance, overdose, and respiratory depression, a significant concern with full opioid agonists [13]. Despite these promising features, findings of a prior Cochrane systematic review of buprenorphine for cancer-related pain management were equivocal [22]. Authors were neither able to conclude that buprenorphine acceptably controlled cancer-related pain nor incidence of side effects and adverse events was lower than full MOR agonists. An updated systematic review of buprenorphine in this population that includes additional study designs is urgently needed.

The primary objective of our systematic review is to address this gap by synthesizing literature on buprenorphine for cancer pain, doing so by expanding the scope to include a broader range of study designs as well as studies published since the 2015 review. Through this approach, we aim to obtain a thorough understanding of buprenorphine’s efficacy, safety, and tolerability in oncology populations. Our findings will elucidate important literature gaps that will inform future research directions and may ultimately impact clinical practice.

## Supplementary Information

Supplementary Material 1.

## Data Availability

Data sharing is not applicable to this article as no datasets were generated or analyzed during the current study.

## Appendix I. PICOTS

### Population

- Patients with a diagnosis of active malignancy or treatment of malignancy, not in remission
- Including children

### Intervention

- Buprenorphine in any form

### Comparator

- Active control (another opioid e.g.)
- Placebo
- No control
- Wait list
- Pre-post
- Case series
- Observational

Exclude: single case report

### Timing

- Variable, but needs to assess pain at least once pre-treatment and once post-treatment using a validated scale

Outcomes:

Primary

- Pain
  - ◦ Intensity/Severity
  - ◦ Interference

Secondary

- Use of breakthrough medication
- Opioid dose reduction
- Side effects
  - ◦ Withdrawal related symptoms
  - ◦ Benign opioid related
- Adverse event
  - ◦ Overdose
  - ◦ Suicide
- Mental health
  - ◦ Depression
  - ◦ Suicidality
  - ◦ Anxiety
- Quality of Life & Function
- Life expectancy
- Illicit drug use
- Healthcare Utilization
  - ◦ Hospitalizations
  - ◦ ER visits

## Appendix II

Full electronic search strategy

### Cochrane CENTRAL

1. MeSH descriptor: [Buprenorphine] this term only
2. ("buprenorphine")
3. (magnogen or temgesic or subutex or transtec or anorfin or bupren or norphin or pentorel or tidigesic or nopan or finibron or brospina or temgesic-nX or buprex or prefin or suboxone or buprenex or buprine or butrans)
4. #1 or #2 or #3
5. MeSH descriptor: [Pain] explode all trees
6. (pain* or nocicept* or neuropath*)
7. #5 or #6
8. MeSH descriptor: [Neoplasms] explode all trees
9. (cancer* or neoplas* or tumor* or carcinoma* or Hodgkin* or nonhodgkin* or adenocarcinoma* or leuk?mia or metasta* or malignan*)
10. #8 or #9
11. #4 and #7 and #10

### Medline

1. buprenorphine/ or buprenorphine.tw. or (magnogen or temgesic or subutex or transtec or anorfin or bupren or norphin or pentorel or tidigesic or nopan or finibron or brospina or temgesic-nX or buprex or prefin or suboxone or buprenex or buprine or butrans).tw
2. exp pain/ or (pain* or nocicept* or neuropath*).tw.
3. exp neoplasms/ or (cancer$ or neoplas$ or tumo$ or carcinoma$ or hodgkin$ or nonhodgk n$ or adenocarcinoma$ or leuk?emia$1 or metasta$ or malignan $ or lymphoma$ or sarcoma$ or melanoma$ or myeloma$ or oncolog$).tw.
4. 1 and 2 and 3
5. (randomized controlled trial or controlled clinical trial).pt. or randomized.ab. or placebo.ab. or drug therapy.fs. or randomly.ab. or trial.ab.
6. 4 and 5
7. buprenorphine/ or buprenorphine.tw. or (magnogen or temgesic or subutex or transtec or anorfin or bupren or norphin or pentorel or tidigesic or nopan or finibron or brospina or temgesic-nX or buprex or prefin or suboxone or buprenex or buprine or butrans).tw.
8. exp pain/ or (pain* or nocicept* or neuropath*).tw.
9. exp neoplasms/ or (cancer$ or neoplas$ or tumo$ or carcinoma$ or hodgkin$ or nonhodgk n$ or adenocarcinoma$ or leuk?emia$1 or metasta$ or malignan $ or lymphoma$ or sarcoma$ or melanoma$ or myeloma$ or oncolog$).tw.
10. 7 and 8 and 9
11. (randomized controlled trial or controlled clinical trial).pt. or randomized.ab. or placebo.ab. or drug therapy.fs. or randomly.ab. or trial.ab.
12. exp animals/ not humans.sh.
13. 10 not 12

### EMBASE

1. 'buprenorphine'/exp OR buprenorphine OR buprenorphine:ti,ab
2. magnogen:ti,ab OR temgesic:ti,ab OR subutex:ti,ab OR transtec:ti,ab OR anorfin:ti,ab OR bupren:ti,ab OR norphin:ti,ab OR pentorel:ti,ab OR tidigesic:ti,ab OR nopan:ti,ab OR finibron:ti,ab OR brospina:ti,ab OR 'temgesic nx':ti,ab OR buprex:ti,ab OR prefin:ti,ab OR suboxone:ti,ab OR buprenex:ti,ab OR buprine:ti,ab OR butrans:ti,ab
3. neoplasm*:ti,ab OR cancer:ti,ab OR cancer*:ti,ab OR neoplas*:ti,ab OR tumo*:ti,ab OR carcinoma*:ti,ab OR hodgkin*:ti,ab OR nonhodgkin*:ti,ab OR adenocarcinoma*:ti,ab OR leukemia*:ti,ab OR metasta*:ti,ab OR malignan*:ti,ab OR lymphoma*:ti,ab OR sarcoma*:ti,ab OR melanoma*:ti,ab OR myeloma*:ti,ab OR oncolog*:ti,ab
4. pain'/exp AND [embase]/lim
5. pain* OR nocicep* OR neuropath*:ti,ab
6. #4 OR #5
7. #1 OR #2
8. #3 AND #6 AND #7
9. random$:ti,ab OR factorial$:ti,ab OR crossover$:ti,ab OR 'cross over':ti,ab OR 'cross-over':ti,ab OR placebo$:ti,ab OR (double$:ti,ab AND adj:ti,ab AND blind$:ti,ab) OR (single$:ti,ab AND adj:ti,ab AND blind$:ti,ab) OR assign$:ti,ab OR allocate$:ti,ab OR volunteer$:ti,ab
10. (animal* OR nonhuman*) NOT human
11. #9 NOT #10
12. #8 AND #11

### Web of Science

1. ALL=(cancer* or neoplas* or tumo* or carcinoma* or hodgkin* or nonhodgkin* or adenocarcinoma* or leuk?emia* or metasta* or malignan* or lymphoma* or sarcoma* or melanoma* or myeloma* or oncolog*)
2. ALL=(pain* or nocicept* or neuropath*)
3. ALL=(buprenorphine)
4. ALL=(magnogen or temgesic or subutex or transtec or anorfin or bupren or norphin or pentorel or tidigesic or nopan or finibron or brospina or temgesic-nX or buprex or prefin or suboxone or buprenex or buprine or butrans)
5. #3 or #4
6. #1 AND #2 AND #5
7. TS=animal*
8. #6 NOT #7

### EBSCO

1. TI buprenorphine OR AB buprenorphine
2. TI (magnogen or temgesic or subutex or transtec or anorfin or bupren or norphin or pentorel or tidigesic or nopan or finibron or brospina or temgesic-nX or buprex or prefin or suboxone or buprenex or buprine or butrans)
3. AB (magnogen or temgesic or subutex or transtec or anorfin or bupren or norphin or pentorel or tidigesic or nopan or finibron or brospina or temgesic-nX or buprex or prefin or suboxone or buprenex or buprine or butrans)
4. TI ( pain* or nocicep* or neuropath* ) OR AB ( pain* or nocicep* or neuropath*)
5. AB((cancer* or neoplas* or tumo* or carcinoma* or hodgkin* or nonhodgkin* or adenocarcinoma* or leuk?emia* or metasta* or malignan* or lymphoma* or sarcoma* or melanoma* or myeloma* or oncolog*))
6. TI ((cancer* or neoplas* or tumo* or carcinoma* or hodgkin* or nonhodgkin* or adenocarcinoma* or leuk?emia* or metasta* or malignan* or lymphoma* or sarcoma* or melanoma* or myeloma* or oncolog*))
7. S1 OR S2 OR S3
8. S5 OR S6

## Abbreviations

GRADE: Grading of Recommendations Assessment, Development and Evaluation
MOR: Mu opioid receptor
OUD: Opioid use disorder
RCT: Randomized controlled trial
